# A 4-Year-Old Boy with an Accidentally Detected Mutation in the RET Proto-Oncogene and Mutation in the Gene Encoding the Ryanodine Receptor1 (RyR1)—Case Report

**DOI:** 10.3390/children10121916

**Published:** 2023-12-12

**Authors:** Magdalena Mierzwa, Małgorzata Blaska, Marek Hamm, Agnieszka Czarniecka, Jolanta Krajewska, Anna Taczanowska-Niemczuk, Agnieszka Zachurzok

**Affiliations:** 1 Department of Pediatrics, Faculty of Medical Sciences in Zabrze, Medical University of Silesia in Katowice, 3 Maja 13-15, 41-800 Zabrze, Poland; d201191@365.sum.edu.pl (M.B.); azachurzok@sum.edu.pl (A.Z.); 2 Nuclear Medicine and Endocrine Oncology Department, M. Sklodowska-Curie National Research Institute of Oncology, Gliwice Branch, 44-101 Gliwice, Poland; marek.hamm@io.gliwice.pl (M.H.); jolanta.krajewska@io.gliwice.pl (J.K.); 3 3rd Department of Oncological Surgery, M. Sklodowska-Curie National Research Institute of Oncology, Gliwice Branch, 44-101 Gliwice, Poland; agnieszka.czarniecka@io.gliwice.pl; 4 Department of Pediatric Surgery, Institute of Pediatrics, Jagiellonian University Medical College, 31-531 Kraków, Poland; anna.taczanowska-niemczuk@uj.edu.pl

**Keywords:** MEN 2B, multiple endocrine neoplasia, RyR1 mutation, short stature, medullary thyroid carcinoma, pediatric patient

## Abstract

Multiple endocrine neoplasia 2B (MEN2B) is a rare syndrome with prevalence estimated at approximately 0.2 per 100,000; it is caused by mutation of the RET proto-oncogene. MEN2B is characterized by early-onset medullary thyroid carcinoma (MTC), ganglioneuromatosis of the aerodigestive tract, marfanoid habitus, ophthalmologic abnormalities, and pheochromocytoma in adulthood. Mutations in the RyR1 gene manifest clinically in congenital myopathies and/or malignant hyperthermia susceptibility. We present a case of a 4-year-old boy with an accidentally detected RET and RyR1 mutations in the course of diagnostic approach of short stature and delayed motor development. Due to a poor and blurred clinical picture of MEN2B syndrome, accompanied by RyR1 mutation symptoms, the diagnostic path was extended. Our patient had no family history of MTC. In the imaging studies of the thyroid gland, no abnormalities were found, whereas the serum level of calcitonin was elevated to 34 pg/mL (N < 5.0). The patient qualified for total thyroidectomy, and the histopathological examination confirmed the diagnosis of MTC. The postoperative serum calcitonin level dropped to normal ranges. This case shows how new genetic diagnostic procedures could be crucial in accidentally diagnosing rare endocrine disease with atypical symptoms, giving an opportunity for relatively early intervention.

## 1. Introduction

Multiple endocrine neoplasia type 2 (MEN2) is a rare, autosomal dominant hereditary cancer syndrome with a prevalence estimated at approximately 0.2 per 100,000; it is caused by germline mutations in the REarranged during Transfection (RET) proto-oncogene. RET encodes a receptor of the tyrosine kinase family that plays a key role in cell differentiation, growth, migration, and survival. This receptor has a crucial role in the development of the nervous system, organs, and tissues derived from the neural crest [1]. MEN2 is divided into two distinct clinical entities: MEN2A and MEN2B [2,3]. M918T mutation in exon 16 of the RET protooncogene is responsible for 95% of cases of MEN2B syndrome. Other RET germline mutations include A833F (<5%), E768D/L790F, V804M/Q781R, V804M/E805 K, and V804M/Y806C [2].

MEN2B is characterized by early-onset medullary thyroid carcinoma (MTC), its aggressive course, pheochromocytoma, and several nonendocrine manifestations such as ganglioneuromatosis of the aerodigestive tract (97%), mostly on the tongue and lips. Constipation, which is often the first symptom, can be caused by ganglioneuromas of the intestinal tract. Musculoskeletal abnormalities such as Marfanoid habitus (73%), hypotonia (27%), pectus excavatum (26%), scoliosis related by hip epiphysiolysis, and foot abnormalities also are often found in MEN2B patients [2,4,5]. Ophthalmic abnormalities include corneal nerve hypertrophy (45%), alacrimia (40%), and neuromas of the eyelid or conjunctiva (19%) [5].

Ryanodine receptor isoform-1 (RyR1) is a major calcium channel in skeletal muscle and is a critical component for excitation-contraction coupling. Mutations in the RyR1 gene manifest clinically as congenital myopathies and/or malignant hyperthermia susceptibility. Mutations in the RyR1 are most often responsible for the occurrence of dominantly inherited central core disease (CCD), recessively inherited multi-minicore disease (MmD), and less often centronuclear myopathy (CNM), congenital fiber-type disproportion (CFTD), and King Denborough syndrome. Ryanodine receptor 1-related myopathies (RyR1-RM) have presented an extensive range of symptoms. They are typically characterized by delayed motor milestones, proximal muscle weakness of the hip girdle, hypotonia, mild facial weakness, ophthalmoplegia, joint laxity/contractures, skeletal deformities, or even moderate to severe respiratory insufficiency [6,7].

## 2. Case Report

We present a 4-year-old male patient with no family history of thyroid cancer who had been under multidisciplinary care since early childhood due to short stature, body weight deficiency, and orthopedic abnormalities. The patient’s mother underwent total thyroidectomy at the age of 29 due to benign nodular goiter. The patient was born in 35 weeks of pregnancy by cesarean section (twin pregnancy 1, birth 1) with a birth weight of 2260 g, birth length of 45 cm, and Apgar score of 10. In the prenatal examinations, clubfeet were shown, confirmed in the examination after childbirth. Moreover, macrocephaly, decreased muscle tone, constipation, weakly developed subcutaneous tissue, and delayed motor development with proper intellectual development were observed. The boy required rehabilitation, Achilles tendon undercuts, and the use of orthopedic shoes. He started walking on his own in the 25 month of life. In the second year of life, the patient was hospitalized in the department of pediatric neurology due to seizures and delayed physical development. In magnetic resonance (MRI) of the brain and electroencephalography (EEG), no abnormalities were found. In addition, the patient was under the care of an ophthalmologist due to astigmatism, a nephrologist due to the widening of the calyx-pelvic system of the left kidney and hypercalcemia, and a cardiologist due to an innocent heart murmur.

From early childhood, the patient’s height remained below the third percentile; his twin sister presented a height within normal ranges. Genetic counseling revealed, in the physical examination, some dysmorphic features, such as macrocephaly, prominent forehead, short neck, abnormalities of oral cavity mucosa in the form of submucosal nodules of lips and tongue, and macroglossia. Moreover, musculoskeletal deformities were observed: scoliosis, pectus excavatum, and hollow foot. Thorough diagnostics was performed to find the cause of the patient’s short stature and nonspecific symptoms. In the genetic testing of comparative genomic hybridization (CGH), there was no genomic change in the tested material. The result of Multiplex Ligation-dependent Probe Amplification (MLPA) for other probably genetic syndromes (Beckwith–Wiedemann Syndrome and Silver–Russell Syndrome) was correct. In the genetic opinion, the patient’s clinical picture did not indicate a specific monogenic disorder. Finally, due to diagnostic difficulties, whole exome sequencing (WES) was performed. The test detected heterozygous pathogenic variant c. 2753T>C (p.Met918Thr) in the RET gene, correlated with MEN2B syndrome and heterozygous pathogenic variant/likely pathogenic variant c. 2505del (p.Pro836LeufsTer48) in the RyR1 gene, correlated with malignant hyperthermia and myopathies inherited both dominant and recessive. After the recognition of MEN2B syndrome, it was necessary to execute immediate diagnostics towards MTC and pheochromocytoma, and the patient was subsequently referred to thyroidectomy.

**Figure 1:**
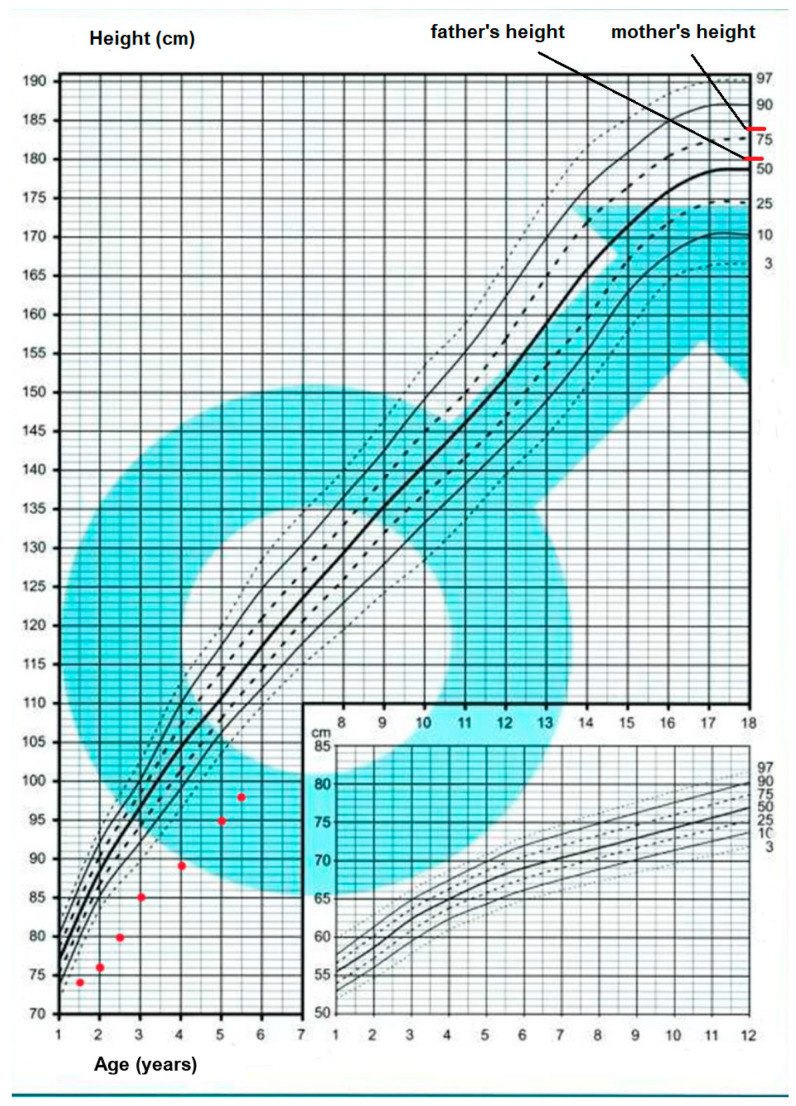
Anthropometric measurements—patient’s growth chart.

The patient was admitted to the pediatric endocrinology department for further examinations. Anthropometric measurements revealed height below the 3rd percentile (89.2 cm, height SDS –3.75) and appropriate body weight (12.3 kg, BMI 15.5 kg/m^2^, 25–50 percentile) (Figure 1). In the basic laboratory tests, the serum level of vitamin D was reduced (22.82 ng/mL N: 30–80). Celiac disease was excluded. Hand X-ray revealed delayed bone age (2.5 years). Growth hormone (GH) levels at the glucagon stimulation test were within the normal ranges for National Polish Standard with a peak of 20.02 ng/mL. Further hormonal tests revealed normal thyroid function (TSH 1.18 uIU/mL; N: 0.27–4.2; fT4 1.1 ng/dL N: 0.93–1.7) and low concentration of insulin-like growth hormone (IGF-1) (16.3 ng/mL; N: 36.6–156.0). Calcitonin serum level was slightly elevated (27.40 pg/mL; N: 0–8.4), while CEA (carcinoembryonic antigen) serum level was within the normal range (2.03 ng/mL; N: 3.8–5.0). Despite the young age of the patient and indication to pheochromocytoma (PHEO) screening beyond the age of 11, to exclude its presence in adrenal glands, a 24-h urine sample was collected. The concentration of methoxyadrenaline, methoxynoradrenaline, and 3-methoxytyramine was within normal ranges. In the abdominal ultrasonography, no abnormalities were detected besides single lymph nodes in the region of the right iliac fossa and rectal fecal masses (patient suffered from chronic constipation). The thyroid ultrasound examination revealed a normoechoic thyroid gland without any nodules. In the neck area, moderately numerous, oval, not enlarged lymph nodes were visible; the largest one 8.5 × 23.0 mm. MRI of the chest and neck was performed, but no suspicious foci were found in the examined area.

The patient and his first-degree family members were referred to the oncology institute. Obligatory genetic testing of the patient’s family members (twin sister and parents) did not reveal the RET mutation, while Sanger’s sequencing of our patient confirmed the diagnosis of RET mutation, showing that RET mutation in our patient was de novo and it was not inherited from his parents. In the repeated thyroid ultrasound, no abnormalities were found, and the control serum calcitonin level remained elevated (34 pg/mL, N < 9.52).

**Figure 2:**
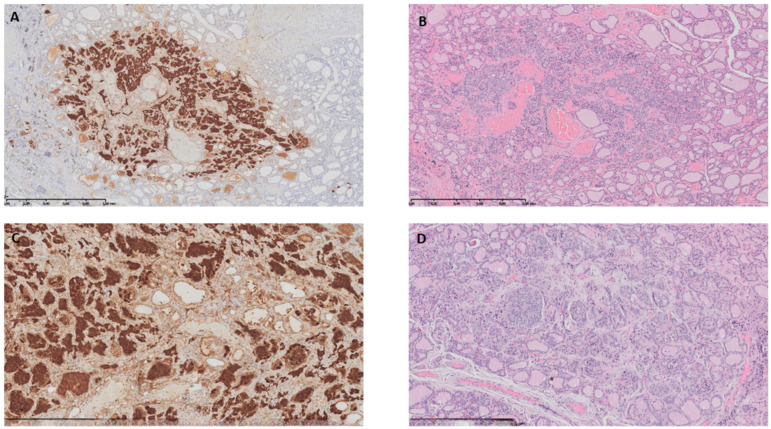
Medullary carcinoma foci in the left (**A**,**B**) and right thyroid lobe (**C**,**D**).

Following the ATA (American Thyroid Association) and Polish national recommendations [8,9], the patient was immediately admitted to the surgery department for total thyroidectomy with lymphadenectomy of the middle compartment and biopsy of lymph nodes of the lateral compartments. Histological examination of the thyroid gland showed two cancer foci measuring 2 mm in the left lobe and 2.2 mm in the right lobe, corresponding to MTC (low grade) (Figure 2). No vascular invasion was found, and the surgical margins were negative. Immunohistochemical expression of MTC marker—calcitonin was positive in the cancer cells. No metastatic structure was found in the assessed lymph nodes. The parathyroid glands and thymus tissue were without neoplastic infiltration.

The surgery was complicated by hypocalcemia. Due to hypothyroidism, levothyroxine supplementation was introduced at a dose of 50 μg per day. One month after thyroidectomy, calcitonin serum level dropped to 2.38 pg/mL (N < 9.52) but remained detectable, while CEA level was normal (2.23 ng/mL; N < 5.0). The patient remains under the care of an endocrinology clinic and oncology institute. The follow-up includes oncologic surveillance (repeated measurements of serum calcitonin and CEA, neck sonography, and other imaging studies, if necessary). and monitoring of thyroid and parathyroid function tests, adjusting supplementation doses according to the results. Recent examinations showed normal calcitonin and thyroid hormone levels. The neck ultrasonography showed no abnormal changes. The patient is 5.5 years old, and his height is still below normal ranges (98 cm < 3 percentile, hSDS −4.57). The diagnostics of short stature has been repeated in the pediatric endocrinology department 1.5 year after previous hospitalization and a low concentration of IGF-1 was confirmed (22.7 ng/mL; N: 36.6–156.0), which can be the underlying cause of the patient’s short stature. In further assessment, disorders of calcium and phosphate metabolism were excluded.

## 3. Discussion

We report the first case of MEN2B patient coexisting with RYR1 mutation found accidentally in WES performed due to unspecific symptoms like decreased muscle tone, delayed neuromotor development, short stature, and other skeletal and ocular abnormalities. The genetic test revealed de novo mutation variant (p.M918T) in the RET gene, which is responsible for 95% of MEN2B cases, and pathogenic/likely pathogenic variant c.2505del(p.Pro836LeufsTer48) in RyR1 associated with myopathies. Overlapping non-specific symptoms made establishing a proper diagnosis in early childhood difficult because they have been found both in MEN2B syndrome and RyR1-RM.

The time of diagnosis determines the prognosis of MEN2B patients. The most potent prognostic factor is the stage of MTC and the presence of extrathyroidal spread [2,5]. Initially, MTC does not cause any alarming symptoms, which leads to a delay in diagnosis. Therefore, it is important to carefully examine the infant patient and notice the extra endocrines features of MEN2B syndrome. However, one should stress that phenotypic MEN2B features are difficult to recognize in such a small child, particularly if there is no positive family history [10]. Brauckhoff et al. [10] reported that the most promising symptoms indicative of MEN2B are ganglioneuroma of the submucous and myenteric plexus, (IGNM)-related constipation, ocular manifestations resulting in tearless crying, less often oral symptoms (thickened “bumpy” lips, neuromas of the tongue, lips, buccal mucosa), and skeletal manifestations (Marfanoid habitus, scoliosis, epiphysiolysis of the hip, pescus excavatum). It confirmed that the chance of remission was higher in patients who had been diagnosed early based on recognition of extra-endocrine symptoms before the development of clinically overt MTC. Hence, the crucial role for survival is early diagnosis of the RET mutation. The importance of them is shown by the MTC outcome of patients who underwent thyroidectomy. The delay in diagnosing of our patient may have been due to the non-specific presentation of extraendocrine manifestations of MEN2B and musculoskeletal system disorders associated with the RyR1 mutation. He has presented clubfeet, scoliosis, pectus excavatum, delayed motor development, and decreased muscle tone. Although hypotonia and foot deformation are also reported in MEN2B, they are both characteristic also for the RyR1 mutation. Moreover, constipation due to ganglioneuromatosis is one of the most frequent extra-endocrine, early features. In our patient this symptom was present, but it was believed to be related to chronic, mild hypercalcemia. The lack of characteristics for MEN2B tall stature and alacrima could have been the primary factor for delaying the proper diagnosis. On the other hand, typical of MEN2B thickened (“bumpy”) lips and neuromas of the tongue and lips were exhibited. Nonetheless, some of these signs could be more pronounced in the subsequent years of life; thus, it is difficult to detect in early childhood. That the diagnosis was not so obvious is also due to the negative family medical history, because the presented patient had de novo RET mutations. According to previous studies, around 80% of M918T carriers have found de novo mutations [2,10]. Taken together, diagnosing this syndrome, although pathognomonic symptoms exist, is still difficult. Elisei et al. [11] described two cases of MEN2B girls who, despite presenting bumpy lips, mucosal neuromas, and skeletal abnormalities from neonatal age, had been diagnosed too late, when the MTC was metastatic. In contrast to this, our patient, thanks to genetic research, had early-stage MTC detected. Therefore, early recognition of specific extra endocrine signs is crucial for establishing proper diagnosis and treatment; however, that is a significant challenge for pediatricians to recognize phenotype of this very rare genetic syndrome.

According to geneticist opinion, our patient’s clinical picture did not indicate any specific monogenic disorder. For this reason, WES was performed, enabling a diagnosis to be established. In recent years, sequencing technologies such as WES and whole-genome sequencing (WGS) have been applied in the diagnostic process. WES and WGS have proved to be disruptive technologies that have rapidly accelerated the discovery of genes underlying Mendelian phenotypes. Moreover, WES is recommended for clinical indications such as multiple birth defects or neurodevelopmental delay when other tests have been uninformative [12]. The use of genetic testing as a first-line test shortens the diagnostic pathway. Detection of the pathogenic mutation enables family counseling, planning, and testing of family members [13]. American College of Medical Genetics and Genomics strongly recommends the use of WES or WGS as the first-line test among pediatric patients with congenital anomalies with onset before the age 1-year, developmental delay, or intellectual disability [14]. In the presented case, WES enabled the diagnosis of the MEN2B-specific mutation together with RyR1 mutation and the performance of thyroidectomy.

Strong genotype-phenotype correlation among MEN2 patients allows us to classify the risk according to their genotype into three groups, according to the MTC aggressiveness, thus defining the timing of prophylactic thyroidectomy [2,8]. The carriers of M918T RET mutation are in the highest MTC risk group, and MTC is detected in 75% of patients in the first year of life [2,5]. In this group, prophylactic thyroidectomy should be performed within the first year of life, even in the first months of life [2,5,8]. Unfortunately, this is largely only possible in MEN2B patients with a positive family history. Castinetti et al. indicated that >80% of M918T RET mutation carriers were cured if thyroidectomy was performed before or at the age of 1. On the contrary, when total thyroidectomy was performed later, only 15% of carriers were cured [5]. However, Brauckhoff et al. [10] presented that MTC diagnosed and surgically treated before the age of 4 allowed for the biochemical cure. In our case, the neck surgery took place after the age of 4 and led to normalization in basal serum calcitonin level without the presence of structural disease in postoperative examinations.

MTC is present in 75% of patients who underwent thyroidectomy within the first year of life [5]. However, due to delays in diagnosis, the median age at thyroidectomy in the Castinetti study was 14 years, and MTC was found in 97% of cases [5]. If MTC is suspected, a measurement of secretory products of thyroid C-cells, the serum calcitonin level (specific MTC tumor marker), as well as CEA (nonspecific MTC marker) should be conducted. Distance metastases are detected initially at diagnosis in around 10% of patients; however, they can appear in approximately a quarter of patients undergoing thyroidectomy during follow up [15]. In our case, the level of calcitonin was elevated but serum CEA level was within the normal range. Moreover, thyroid ultrasound revealed only slightly enlarged lymph nodes, without distinguishing structures, and no suspicious foci were found in MRI of the chest and neck. Nevertheless, postoperative histological examination of the thyroid gland confirmed two MTC foci. Fortunately, total thyroidectomy, although performed after 1 year of age, resulted in normalization of serum calcitonin level and the absence of structural disease.

In our patient, WES also revealed a missense RyR1 mutation associated with MmD, CNM, and King Denborough syndrome core myopathies (dominant inheritance). The prevalence of RyR1-related myopathies in the pediatric population is estimated to be 1:90 000, which are inherited in both autosomal dominant and recessive manners and de novo [7]. The spectrum of symptoms in these disorders is broad. Patients typically present proximal muscle weakness, hypotonia, mild facial weakness, joint laxity, scoliosis, kyphoscoliosis, ophthalmoplegia, and moderate to severe respiratory insufficiency. In the literature, the case report of a 6 month old boy with the same mutation in RYR1 gene as our patient was described. The boy presented respiratory disorders, hypomic face, pectus excavatum, cryptorchidism, severe muscle hypotonia, and hydrocephalus and a variant of Dandy Walker’s in MRI of the brain. The genetic testing revealed pathogenic variant of c.2505del p.Pro836Leufs*48 in RYR1. Muscle biopsy was performed. According to biopsy data and genetic analysis, the central core disease associated with malignant hyperthermia was detected [16]. In our patient, neonatal hypotonia, delayed motor development, and spinal and limb deformities have been observed. However, there is no occurrence of ophthalmoplegia or respiratory failure, so it seems that the muscular disorders are of a mild nature. Consequently, a muscle biopsy was not performed on our patient, making it impossible to identify the subtype of RyR1-RM. Missense mutation RyR1 has been linked to malignant hyperthermia (MH), where exposition to halogenated inhalation anesthetics (e.g., sevoflurane, desflurane) or depolarizing blockers of skeletal muscle contraction (succinylcholine) can lead striking increase in end-tidal carbon dioxide, intense production of heat, tachycardia, unstable hemodynamics, lactic acidosis, and skeletal muscle rigidity. That episode can be lethal if it is not quickly treated with the administration of dantrolene (muscle relaxant) drugs [6,7]. With this knowledge and proper anesthetic management, there were no complications in our patient during thyroidectomy. Currently, no specific treatments for RyR1-related disorders are available. However, therapeutic approaches have been increasingly explored. Clinical trials are currently underway for the effectiveness ryanodine channel complex stabilizer compound S48168/ARM210; moreover, fluorescence energy transfer (FRET)-based high-throughput screening (HTS) assay and ER Ca^2+^-based assays are encouraging methods for identifying novel compounds as RyR1-inhibitors. It is worth pointing out that gene therapies appear to be promising methods for the future treatment of RyR1-related myopathies [17]. However, the mild clinical course observed in our patient makes it probable that he would not need such sophisticated treatment for RyR1-related disorders.

Our patient has short stature without GH deficiency but with low IGF1, uncharacteristic of Marfanoid habitus related to MEN2B. Nevertheless, numerous cases of MEN2B patients with short stature are described in the literature [18,19,20,21,22]. In some cases, GH deficiency was discovered [20,22]. Redlich et al. [22] presented one child treated with recombinant human GH (rhGH) who showed catch-up growth. The height below the 3rd percentile without any further growth delay progression in our patient is in agreement with the literature description on short-stature MEN2B children. The patient has presented a huge difference in height compared to his twin sister and has been outside the predicted height range related to parents’ heights. He is not GH deficient and rhGH therapy is not indicated. However, his short stature could be related to low IGF1 secretion, and IGF1 analogue, mecasermin, may be the therapeutic option. The RET protooncogene encodes a receptor tyrosine kinase that plays a key role during embryonic development of the nervous and urogenital systems and tissues derived from the neural crest, e.g., the C-cells of the thyroid gland, adrenal medulla [1,2]. Nowadays we do not know the impact of rhGH and other growth factors (mecasermin) therapy on the risk of cancers in RET mutation carriers, especially with its clinical manifestation. There is no data on the long-term safety and efficiency of growth promoting therapy in MEN2B patients; this is a consequence of the small number of MEN2B patients treated with growth hormone and no MEN2B patient who was treated with mecasermin.

To the best of our knowledge, it is the first reported case of co-occurrence of MEN2B syndrome and RyR1 mutation in the literature. This example proves that rare endocrinological syndromes with malignant potential should be taken into consideration even if atypical symptoms are observed. Lack of standard manifestations can delay the proper diagnosis and worsen the prognosis. This case also shows how new genetic diagnostic procedures could be important in accidentally diagnosing rare endocrine diseases, giving opportunity to early intervention.

We are aware of some limitations of our study. There is a short-term follow-up; we are not able to provide long-term observation, longer than 5 years, because the age of our patient at this moment is 5 years, and WES diagnosis was established at the age of 3 y. Therefore, we could not see how the consequences of mutation influence the clinical outcome. Moreover, at this moment we have not received the results of genetic analysis of RYR1 mutation in first degree relatives; therefore, we do not know if the mutation is de novo or inherited from the parents, which is important for determining the risk of the disease in the proband’s twin sister and offspring. Such analysis is planned in the near future.

## Data Availability

No new data were created or analyzed in this study. Data sharing is not applicable to this article.
